# VALIDATION OF AN OPTICAL METHOD FOR MEASURING THE GAP BETWEEN SUTURED TENDON STUMPS

**DOI:** 10.1590/1413-785220263404e301170

**Published:** 2026-07-24

**Authors:** Luiz Sorrenti, Elcio Koodiro Yoshida, Antonio Isidoro de Sousa, César Augusto Martins Pereira, Hugo Alberto Nakamoto, Rames Mattar

**Affiliations:** 1 Universidade de Sao Paulo, Faculdade de Medicina, Hospital das Clinicas, (HC-FMUSP), Instituto de Ortopedia e Traumatologia, Hand Surgery and Reconstructive Microsurgery Group, Sao Paulo, SP, Brazil.

**Keywords:** Tendons, Tendon Transfer, Biomechanical Phenomena, Suture Techniques, Tendões, Transferência Tendinosa, Fenômenos Biomecânicos, Técnicas de Sutura

## Abstract

**Objective:**

This study aims to investigate the accuracy and precision of an optical measurement method based on backlight video analysis for measuring gap distances between tendon stumps in a rigid tendon model.

**Methods:**

A rigid 3D-printed tendon model was sutured and subjected to traction using a universal mechanical testing machine. The gap between stumps was filmed in frontal and lateral planes with backlight illumination and a simple mirror system. A custom software program calculated gap distances from the recorded videos and the optical measurements were compared with mechanical testing machine measurements to assess precision and accuracy.

**Results:**

The mean difference (bias) in the frontal plane was -0.01 mm (SD: 0.01 mm) and in the lateral plane was 0.04 mm (SD: 0.03 mm). Bland-Altman analysis showed limits of agreement (LoA) of 0.04 mm in the frontal plane and 0.06 mm in the lateral plane. Measurements between planes showed a very strong correlation with a Spearman's rho correlation test of 1.000 (p<0.001).

**Conclusion:**

The optical method showed errors on the order of hundredths of a millimeter and proved to be both precise and accurate, validating a new method for gap measurement in biomechanical tendon studies. **Level of Evidence IV; Experimental Validation Study.**

## RESUMO

**Objetivo:**

Este estudo tem como objetivo investigar a acurácia e a precisão de um método de mensuração óptica baseado na análise de vídeo com retroiluminação para medir as distâncias do afastamento entre cotos tendíneos em um modelo tendíneo rígido.

**Métodos:**

Um modelo tendíneo rígido impresso em 3D foi suturado e submetido à tração em uma máquina universal de ensaios mecânicos. O espaço entre os cotos foi filmado nos planos frontal e lateral com retroiluminação e um sistema simples de espelho. Um software personalizado calculou as distâncias do espaço a partir dos vídeos gravados, e as mensurações ópticas foram comparadas com as mensurações da máquina de ensaios mecânicos para avaliar a precisão e a acurácia.

**Resultados:**

A diferença média, ou viés, no plano frontal foi de -0,01 mm (DP: 0,01 mm) e, no plano lateral, de 0,04 mm (DP: 0,03 mm). A análise de Bland-Altman apresentou limites de concordância (LoA) de 0,04 mm no plano frontal e de 0,06 mm no plano lateral. As mensurações entre os planos apresentaram correlação muito forte, com coeficiente rho de Spearman de 1,000 (p<0,001).

**Conclusão:**

O método óptico apresentou erros da ordem de centésimos de milímetro e demonstrou ser preciso e acurado, validando um novo método para mensuração do espaço em estudos biomecânicos de tendões. **Nível de Evidência IV; Estudo de Validação Experimental.**

## INTRODUCTION

Tendon is a tissue formed mainly by collagen (65-80%), elastin (1-2%) produced by tenocytes, surrounded by a proteoglycan matrix responsible for transmitting muscle force to joints and bones.^1^ It has the capacity to adapt to mechanical loads by modifying its structure, composition and mechanical properties^2,3^, however, its overload can result in acute or chronic injuries.^4^ Injuries of flexor tendons of the fingers often result in functional disability^5^ and it is known that resistant suturing techniques to allow early active mobilization are essential for healing, prevention of adhesion formation and better results.^6^ Despite advances in suturing techniques and post-operative rehabilitation, an important complication is the formation of a tendon gap, the distance between the edges of the two tendon stumps that are joined by the surgical suture, when subjected to traction forces, which can lead to poor functional results after tendon repair.^7^

A gap greater than 2 mm after repair of the flexor digitorum profundus tendon significantly increases the resistance to sliding, leading to a higher risk of adhesion and the need for a future tenolysis.^6-8^ Linnanmäki et al.^9^ concluded that, when the suture is subjected to repeated stress, there is a suture fatigue point coinciding with the beginning of tendon plastic deformation that predicts the formation of gaps and then increases the risk of failure if repeated stress is maintained. Furthermore Gelberman et al.^10^ established that the formation of a 3 mm gap is detrimental to the gain in strength and stiffness in tendon healing, using a canine model.

The formation of a gap in the tendon suture compromises the quality of the tenorrhaphy and we have observed the absence of new methods for measuring the formation of this gap.^7,11^ Therefore, there is a need to update the methods for measuring the gap between tendon stumps in experimental tests, seeking greater precision of the measured distance between tendon stumps. Greater precision in this analysis can improve the quality of existing techniques for tendon suturing. It is expected that there will be a direct benefit to patients, through the standardization of the recording and evaluation of the gap in trials, research and development of new surgical techniques. The aim of this study is to validate the accuracy of the optical method for measurement of the distance, in the frontal and lateral planes, between the stumps of a 3D-printed tendon model in biomechanical experiments with the universal mechanical testing machine.

## METHODS

### Model

**Figure 1:**
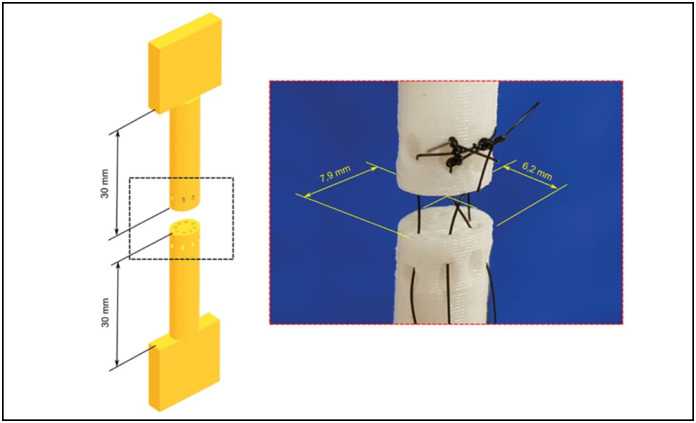
3D-printed tendon model - Stump faces have eight holes for the tendon suture.

The study obtained gap measurements of the tendon stump model in the frontal and lateral planes by filming it with the help of a mirror reflecting the lateral face and backlighting the model in both planes. The Kratos K5002 universal mechanical testing machine (Kratos Equipamentos Industriais Ltda., São Paulo, São Paulo, Brazil) was used to precisely separate the tendon stump and acquire the distance of the movement. A Canon EOS Rebel T2i digital camera with a Canon EF-S 18-135mm f/3.5-5.6 IS STM lens (Canon Inc., Japan) recorded the footage. The images were processed using specific software developed for the study by the authors. The tendon stump model was made with the shape of an elliptical cylinder and split orthogonally to reproduce the faces of the stumps of a ruptured tendon (Figure 1). It was printed using a 3D Machine One 3D filament printer in white polylactic acid (PLA) plastic. The rigid plastic material was chosen so that there would be no significant deformation of the material during traction of the stumps by the universal mechanical testing machine.

The model was based on the thickness of the flexor digitorum profundus tendon of the adult human hand and has an elliptical cross-section with a major width of 7.9 mm and a minor width of 6.2 mm and a length (longitudinal axis) of 30 mm for each part of the stump.^12^ Eight holes were made on the split sides of the stumps to allow the surgical sutures to pass through, simulating the tenorrhaphy of the tendon stumps repair (Figure 1).

**Figure 2:**
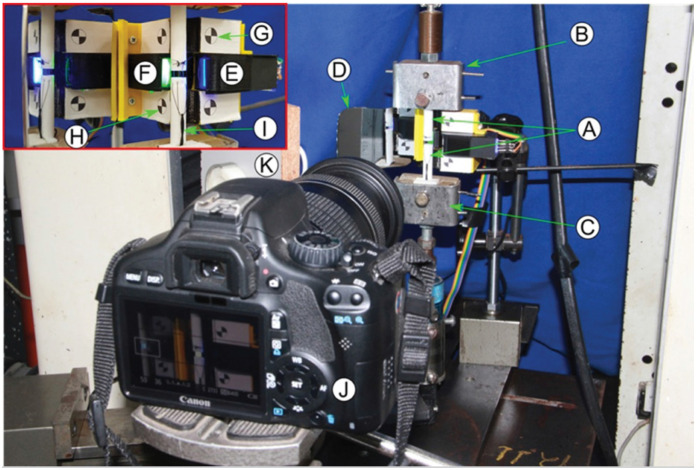
Assembly of the measuring system - Tendon model (A), fixation claws (B and C), metallic mirror (D), backlighting light sources (E and F), templates (G and H), camera (J) and light source (K). The sutures were tensioned with an elastic band (I) attached to the lower clamp (C).

The two parts of the tendon model were fixed to the universal mechanical testing machine by rigid clamps ensuring that the axis of the displacement aligned with the longitudinal axis of the stumps and no other movement could occur. The machine recorded a precise distance and time during the tests (Figure 2A, B and C). The two parts of the stump were connected simulating a four-strand tenorrhaphy using black nylon suture No. 4-0. The suture was tensioned with an elastic band and attached to the base of the model without causing excessive traction on the stump during its separation (Figure 2I).

To start the test, the faces of the model's stumps were brought together, setting the initial gap at zero. To record the gap optically in the frontal and lateral planes of the tendon model, the camera was positioned on a tripod at the same height as the model (Figure 2J). The framing of the footage was in landscape, with the tendon model centered, obtaining the frontal plane of the stumps, and on the left side the mirror image of the lateral face of the model, obtaining the lateral plane of the stumps (Figure 2D). An Indusbello IBM-001 metallic mirror (Indusbello, Londrina, Paraná, Brazil) was used so that the image would not be double reflected like a regular glass mirror.

**Figure 3:**
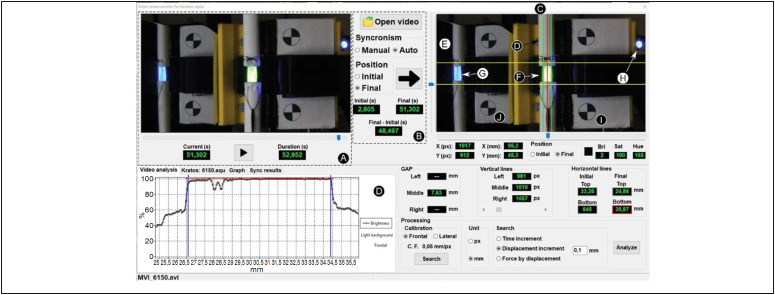
Measurement software - Video controls (A), position and synchronization controls (B). On the right (C) the tendon model video (D) and the lateral mirrored image of the tendon model (E), the synchronism LED (H), frontal (I) and lateral (J) template, frontal (F) and lateral (G) backlight source. The graph (D) represents the brightness (y-axis, %) corresponding to the red vertical line bounded by the two yellow horizontal lines (x-axis, mm).

The gap measurements were obtained by using printed templates of known distances included in the same planes as the filmed image of interest (Figure 2G, H). The distances between the centers of the templates were previously obtained by the Deltronic DV 114 profile projector, with a linear accuracy of 0.005mm (Deltronic, Santa Ana, California, EUA), which allows the filming to be calibrated (Figure 3). Two LED projectors were positioned orthogonally to backlight the model to increase the luminous contrast with the passage of light through the gap formed when the stumps were separated. The front backlight of the stump was green and the mirrored side backlight was blue.

To ensure that the displacement and time data from the universal mechanical testing machine was synchronized with the filming, an LED was included which turned on when the displacement was made by the machine, marking the start of the movement when it turned on and the end when it turned off (Figure 3H).

Computer software based on the Free Pascal language was developed by the author within the Typhon integrated development environment (Video 1S - https://youtu.be/wAKF_sRU-pE). This software identifies the gap as the region between the stumps backlit by the projector during filming. The software used the fixed templates to determine the gap in millimeters. Each test was carried out at a speed of 10 mm/min until a total movement of 6 mm was recorded by the universal mechanical testing machine, with a displacement accuracy of hundredths of a millimeter. Ten tests were repeated with the tendon model. The displacement information measured by the machine was recorded using the Lynx ADS 2000 data acquisition system with a displacement resolution of 0.01 mm (Lynx Tecnologia Eletrônica Ltda., São Paulo, São Paulo, Brazil).

In order for the software to automatically take measurements of the gap in the tendon model, the user must set up the following within the program itself: synchronism, selection of the analysis area (position), calibration and selection of the search criteria for the gap.

Software settings:

Synchronism: Time synchronization of the footage with the data obtained by the universal mechanical testing machine. In the auto option, the user enters the approximate location of the synchronism LED in the image to recognize the start and end of the movement.

Selection of the analysis area (position): delimitation of the image for the evaluation and analysis of the gap formed, by positioning the green vertical lines and the yellow horizontal lines.

Calibration: In the calibration process, the software asks the user for the approximate location of the center of each template seen in the footage, and the centers of the templates are automatically identified. Calibration is defined as the ratio of the known distance of the template to the distance in pixels of the points found.

Gap search criteria: The software determines the search criteria, which can be by time increment; displacement increment according to the progression of the universal mechanical testing machine, a criteria used in the validation of the model; and by predefined gap (Force by GAP) with the recognition of the increase in the gap in the filming, interesting in comparative studies.

### Methods of Assessment

**Figure 4:**
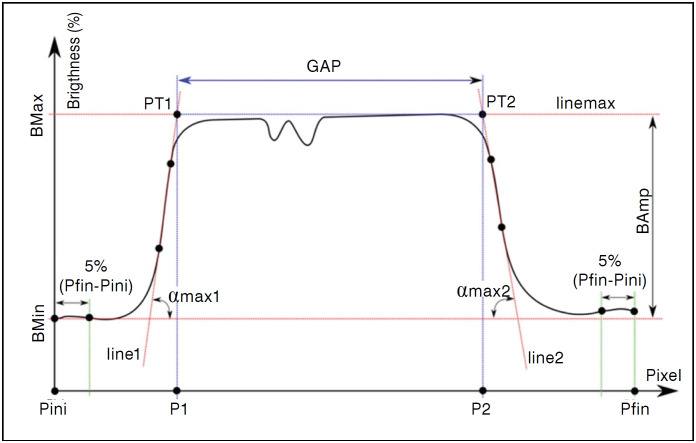
Graph representing the gap distance of the backlit tendon model - It shows the distribution of brightness measured vertically on the image between the initial (Pini) and final (Pfin) points.

The gap measurement carried out by the software is based on the sudden change in brightness caused by the contrast between the stump model and the background at the end of the stumps (Figure 4), and this interval is transformed into millimeters through the software's calibration with the fixed template distances included. The software calculates the average gap in the interval bounded by the green vertical and yellow horizontal lines, intervals manually selected before the start of the filming process (Figure 3), for both frontal and lateral analysis, ensuring that the gap region is contained.

### Statistical Analysis

This study compared measurements obtained from footage processing against the measurements recorded by a universal mechanical testing machine, utilizing the Lynx ADS 2000 data acquisition system during the stumps separation. Descriptive statistics, including the mean difference (bias), median, standard deviation (SD), interquartile range (IQR), and standard error of the mean (SEM), were calculated from the differences between footage measurements and data acquisition system measurements. These statistics were reported for each of the ten individual runs and for the aggregate 600 data pairs in both the frontal and the lateral planes, with no missing values. A significance level (α) of 0.05 and a statistical power (1−β) of 0.80 were adopted for this study.

The Bland-Altman analysis^13^ was the primary method for assessing limits of agreement (LoA). These graphical and statistical plots differences against averages to identify systematic bias and calculates the 95% LoA, within which 95% of differences are expected to fall. The adequacy of the sample size (600 pairs) for the Bland-Altman analysis was confirmed using the method proposed by Lu et al.^14^ This retrospective assessment determined the maximum allowed difference with the given sample size. Spearman's rho correlation test was performed to assess the relationship between the frontal and lateral plane measurements.

**Figure 5:**
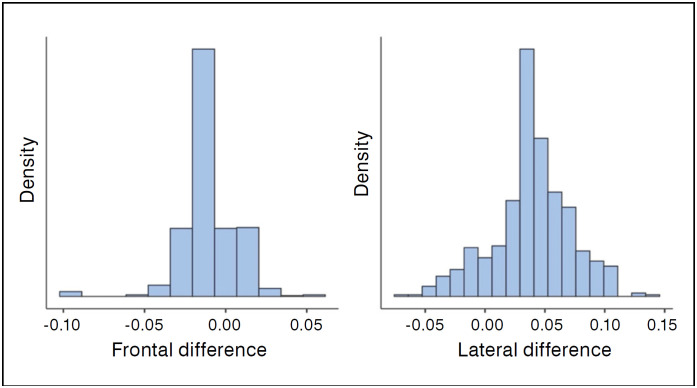
Frontal and lateral differences distribution - Graphics of differences of the measured gap and the displacement by the Kratos K5002 universal mechanical testing machine. With Shapiro-Wilk test with p < 0.05. Frontal plane on the left and lateral plane on the right.

Prior to assessing the agreement between the two measurement methods, the normality of the differences was evaluated using the Shapiro-Wilk test. For both frontal and lateral plane differences, the p-value was less than 0.05, indicating a non-normal distribution of the data (Figure 5). Although Bland-Altman analysis usually uses normally distributed data, it was employed in this study to determine the LoA to provide a descriptive measure of agreement, with the understanding that the interpretation of these limits acknowledges the non-normal distribution of the differences. Given the minimal variation observed between the methods, the standard deviation (SD), interquartile range (IQR), and standard error of the mean (SEM) were reported with three decimal places, reflecting a precision less than the data acquisition system's limit.

The methodology proposed by Lu et al.^14^ was utilized to determine the maximum allowable difference in a scenario of 600 tests. This approach is usually used to evaluate the adequacy of the Bland-Altman analysis sample size by pre-determining alpha, beta, and a maximum acceptable difference. In this study, a retrograde analysis was performed to assess these parameters of a maximum acceptable difference for the aggregate test.

## RESULTS

Ten runs were performed with 60 measurements and a total of 600 pairs of measurement were recorded. The mean difference or bias of all data in the frontal plane was -0.01 mm (SD: 0.01 mm) and in the lateral plane was 0.04 mm (SD: 0.03 mm) (Table 1). It can be noted that the difference between the measurement performed by the method and the measurement obtained by the universal mechanical testing machine was on the order of hundredths of a millimeter in the frontal and lateral planes.

**Table 1:** Descriptive data of differences of the measured gap and the displacement by the Kratos K5002 universal mechanical testing machine.

	Frontal Plane		Lateral Plane	
Run	Difference Mean ± SD; Difference Median (IQR)	SEM	Difference Mean ± SD; Difference Median (IQR)	SEM
1	-0.03 ± 0.012; -0.03 (0.010)	0.002	0.03 ± 0.031; 0.04 (0.030)	0.004
2	-0.02 ± 0.014; -0.02 (0.010)	0.002	0.04 ± 0.032; 0.04 (0.030)	0.004
3	-0.01 ± 0.011; -0.01 (0.010)	0.001	0.04 ± 0.027; 0.04 (0.033)	0.004
4	-0.01 ± 0.015; -0.01 (0.000)	0.002	0.04 ± 0.032; 0.04 (0.033)	0.004
5	-0.01 ± 0.015; -0.01 (0.010)	0.002	0.04 ± 0.031; 0.05 (0.040)	0.004
6	-0.01 ± 0.009; -0.01 (0.000)	0.001	0.03 ± 0.032; 0.04 (0.040)	0.004
7	-0.03 ± 0.013; -0.03 (0.010)	0.002	0.03 ± 0.030; 0.03 (0.043)	0.004
8	0 ± 0.012; 0 (0.010)	0.002	0.05 ± 0.029; 0.06 (0.033)	0.004
9	0.02 ± 0.010; 0.02 (0.010)	0.001	0.06 ± 0.031; 0.06 (0.042)	0.004
10	-0.02 ± 0.013; -0.01 (0.010)	0.002	0.04 ± 0.038; 0.04 (0.042)	0.005
All	-0.01 ± 0.018; -0.01 (0.020)	0.001	0.04 ± 0.033; 0.04 (0.040)	0.001

Standard deviation (SD); interquartile (IQR); Standard Error Mean (SEM).

**Figure 6:**
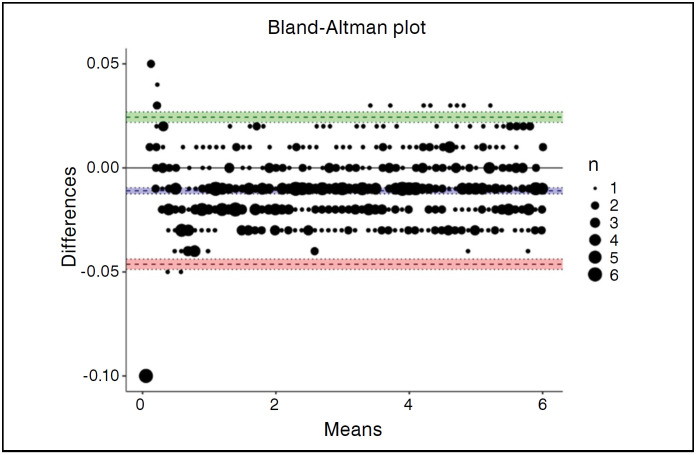
Bland-Altman plot of frontal plane measurements - Bland-Altman plot with upper limit of agreement (LoA) and confidence intervals of 95% in green, lower LoA and confidence intervals in red and bias and confidence intervals of 95% in blue. Circle size represent the density.

**Figure 7:**
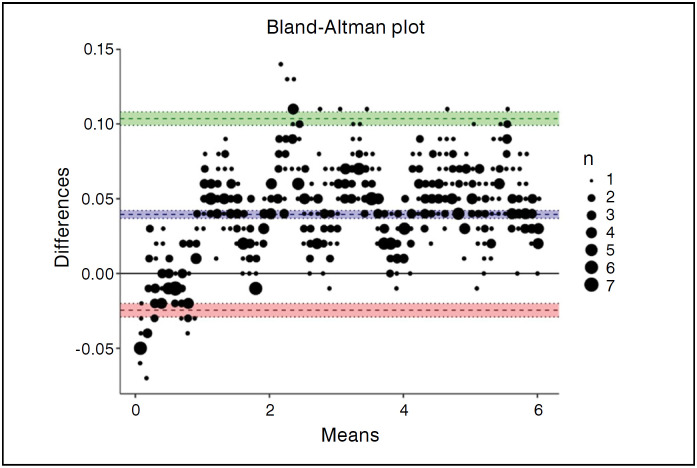
Bland-Altman plot of lateral plane measurements - Bland-Altman plot with upper limit of agreement (LoA) and confidence intervals of 95% in green, lower LoA and confidence intervals in red and bias and confidence intervals of 95% in blue. Circle size represent the density.

To describe the agreement between the collected data and universal mechanical testing machine, Bland-Altman analysis was performed using the Jamovi Version 2.6 statistical software (Jamovi, Sydney, Australia) with blandr module. The Bland-Altman analysis gave a frontal plane LoA of 0.04, upper LoA of 0.02 mm (Table 2, Figure 6) and lower LoA of -0.05 mm and a lateral plane LoA of 0.06, upper LoA of 0.10 mm and lower LoA of -0.02 mm (Table 3, Figure 7), and it was considered precise by the authors. With 600 tests and predetermined alpha and beta, the Lu et al. analysis showed a maximum allowed difference (Δ) of 0.05 mm for the frontal plane and a value of 0.11 mm for the lateral plane. The Med Calc® software (MedCalc Software Ltd, Ostend, Belgium) was used to perform this test. The Bland-Altman analysis was in agreement with the Lu et al. test^14^, with lower values.

**Table 2:** Bland-Altman analysis of frontal plane measurements with 95% confidence interval of limit of agreement.

		95% Confidence Interval	
	Estimate	Lower	Upper
Bias (n = 600)	-0.011	-0.012	-0.010
Lower limit of agreement	-0.046	-0.049	-0.044
Upper limit of agreement	0.024	0.022	0.027

**Table 3:** Bland-Altman analysis of lateral plane measurements with 95% confidence interval of limit of agreement.

		95% Confidence Interval	
	Estimate	Lower	Upper
Bias (n = 600)	0.040	0.037	0.042
Lower limit of agreement	-0.025	-0.029	-0.020
Upper limit of agreement	0.104	0.099	0.108

These data show that the average gap measurement in the lateral reflected plane was not as accurate and precise as that in the frontal plane, with an average difference of 0.05 mm and with the standard deviation being small as 0.03 mm between the planes. The Spearman rho's correlation test was very strong and with a significant correlation (1.000; p<0.001). No major complications or unexpected failures occurred during testing.

## DISCUSSION

The determination of the precise distance between tendon stumps during biomechanical strength testing is essential for the development of a surgical technique that allows early active movement in the rehabilitation of tendon injuries, especially in the flexors of the fingers ^15^ But several mechanisms have been described in the literature for measuring the distance formed between tendon stumps in comparative tests of different tendon suturing techniques and most are imprecise, on the order of a millimeter, or very expensive and complex.^6,12^

Zhao et al.^7^ and Lee et al.^16^ recorded the formation of the gap using a digital video camera at a speed of 30 frames per second, associated with a micrometer ruler placed parallel and coplanar to the tendon. Croog et al.^17^ used digital images at 5 frames per second, filmed using a ruler along the tendon. Lawrence et al.^18^ used visual inspection with the aid of magnifying glasses with 2 to 3 times magnification to determine the moment when the gap occurs. Wong et al.^19^ use photographic images associated with a ruler. In all cases, the measurements can suffer great inter-observer and even intra-observer variation.

In the case of Linnanmäki et al.^9^, the gaps were measured statically after load application using camera photos, in comparison with photos taken previously, using computer software, but without the use of backlight for measurement, which does not prevent measurement errors due to changes in brightness and shadows. Yang et al.^20^ compared the force required to generate a 2 mm gap in different types of sutures and used a caliper to perform the measurements, which can lead to variations and inaccuracies in the results.

No methods found in the literature associate the measurement of the gap in two orthogonal planes at the same time, frontal and lateral, through the analysis of images of the tendon backlighted with a light source. These methods use image analysis with the tendon illuminated in a single plane (frontal) and require a more sophisticated image analysis to detect the gap, since factors such as lighting, color of the object and of the background can interfere in the process.

In addition, there is an established correlation between the number of strands and the tension force of a tendon repair.^19^ Therefore, sutures with different numbers of strands should be compared. In measurements taken with tenorrhaphy sutures, it was noted that they did not interfere with the gap measurements. The new method allows comparisons to be made between sutures with different numbers of passes, without interfering with the gap measurements. It is also possible in tests involving suture resistance, to correlate the size of the gap formed and the force required for it with the universal mechanical testing machine. Validation of the measurement system using a rigid plastic model and rigid clamps without resistance was necessary because the use of natural tendon would not allow direct correspondence between the data from the displacement meter of the universal mechanical testing machine and those recorded by the camera, due to their intrinsic elasticity and deformation before separation occurs. Also, for this reason, an isolated analysis of the measurement of the separation of the machine's data is not useful in biomechanical tests with natural tendons subjected to repair. These validations used a rigid model and future extrapolation to biological tissues should be cautious, considering tissue elasticity and deformation.

Validation of the method allows for full reliability in the measurements provided by the images and analyzed by the software, creating an accurate measurement method on the order of hundredths of a millimeter. The validation method is reproducible, low-cost, and capable of simultaneous dual-plane evaluation.

## CONCLUSION

The optical method offers a reliable and accurate tool for measuring gap distances between tendon stumps on the order of hundredths of a millimeter. It is a promising technique for biomechanical research involving natural tendon repair evaluation.

## Data Availability

The authors confirm that the data supporting the findings of this study are available within the article. In addition, the datasets used and/or analyzed during the current study are available from the corresponding author upon reasonable request.

## References

[1] Kannus P (2000). Structure of the tendon connective tissue. Scand J Med Sci Sports.

[2] Wang JH (2006). Mechanobiology of tendon. J Biomech.

[3] Wang JH, Guo Q, Li B (2012). Tendon biomechanics and mechanobiology--a minireview of basic concepts and recent advancements. J Hand Ther.

[4] Kannus P, Paavola M, Paakkala T, Parkkari J, Järvinen T, Järvinen M (2002). Pathophysiology of overuse tendon injury. Radiologe.

[5] Gundlach BK, Zelouf DS (2023). Flexor tendon reconstruction. Hand Clin.

[6] Tang JB, Lalonde D, Harhaus L, Sadek AF, Moriya K, Pan ZJ (2022). Flexor tendon repair: recent changes and current methods. J Hand Surg Eur Vol.

[7] Zhao C, Amadio PC, Tanaka T, Kutsumi K, Tsubone T, Zobitz ME (2004). Effect of gap size on gliding resistance after flexor tendon repair. J Bone Joint Surg Am.

[8] Angeles JG, Heminger H, Mass DP (2002). Comparative biomechanical performances of 4-strand core suture repairs for zone II flexor tendon lacerations. J Hand Surg Am.

[9] Linnanmäki L, Göransson H, Havulinna J, Sippola P, Karjalainen T, Leppänen OV (2018). Gap formation during cyclic testing of flexor tendon repair. J Hand Surg Am.

[10] Gelberman RH, Boyer MI, Brodt MD, Winters SC, Silva MJ (1999). The effect of gap formation at the repair site on the strength and excursion of intrasynovial flexor tendons. An experimental study on the early stages of tendon-healing in dogs. J Bone Joint Surg Am.

[11] Haddad R, Peltz TS, Lau A, Bertollo N, Nicklin S, Walsh WR (2010). The relationship between gap formation and grip-to-grip displacement during cyclic testing of repaired flexor tendons. J Biomech.

[12] Mao WF, Wu YF, Zhou YL, Tang JB (2011). A study of the anatomy and repair strengths of porcine flexor and extensor tendons: are they appropriate experimental models?. J Hand Surg Eur Vol.

[13] Altman DG, Bland JM (1983). Measurement in medicine: the analysis of method comparison studies J R Stat Soc Ser D (The Statistician).

[14] Lu MJ, Zhong WH, Liu YX, Miao HZ, Li YC, Ji MH (2016). Sample size for assessing agreement between two methods of measurement by Bland-Altman method. Int J Biostat.

[15] Pan ZJ, Pan L, Xu YF, Ma T, Yao LH (2020). Outcomes of 200 digital flexor tendon repairs using updated protocols and 30 repairs using an old protocol: experience over 7 years. J Hand Surg Eur Vol.

[16] Lee HI, Lee JS, Kim TH, Chang SH, Park MJ, Lee GJ (2015). Comparison of flexor tendon suture techniques including 1 using 10 strands. J Hand Surg Am.

[17] Croog A, Goldstein R, Nasser P, Lee SK (2007). Comparative biomechanic performances of locked cruciate four-strand flexor tendon repairs in an ex vivo porcine model. J Hand Surg Am.

[18] Lawrence TM, Davis TR (2005). A biomechanical analysis of suture materials and their influence on a four-strand flexor tendon repair. J Hand Surg Am.

[19] Wong YR, Lee CS, Loke AM, Liu X, Suzana MJ I, Tay SC (2015). Comparison of flexor tendon repair between 6-strand Lim-Tsai with 4-strand cruciate and Becker technique. J Hand Surg Am.

[20] Yang W, Li J, Su Y, Liang W, Ren Y, Dong Y (2021). A modified flexor tendon suture technique combining kessler and loop lock flexor tendon sutures. Clinics (Sao Paulo).

